# Presumed clomiphene-induced optic neuropathy: A case report

**DOI:** 10.18502/ijrm.v19i5.9257

**Published:** 2021-06-23

**Authors:** Yousef Alizadeh, Zahra Moravvej, Yaser Khakpour, Ebrahim Azaripour, Mitra Akbari, Reza Soltani-Moghadam

**Affiliations:** Department of Eye, Eye Research Center, Amiralmomenin Hospital, School of Medicine, Guilan University of Medical Sciences, Rasht, Iran.

**Keywords:** Clomiphene citrate, Optic neuropathy, Visual acuity, Ischemia.

## Abstract

**Background:**

Clomiphene citrate is an estrogen receptor ligand with mixed agonistic–antagonistic properties used for the treatment of female and male infertility. Various visual disturbances and several irreversible visual outcomes have been associated with clomiphene citrate. In this report, we present a patient with presumed clomiphene-induced optic neuropathy.

**Case:**

A 33-yr-old man with acute visual loss of the right eye was referred to Amiralmomenin Hospital, Rasht, Iran in November 2018. His only medication was clomiphene citrate 100 mg daily, taken for 2 wk for fertility issues. The patient presented with a sudden decrease of visual acuity in the right eye on the 14${}^{\mathrm{th}}$ day of starting the treatment and subsequently developed complete loss of inferior visual field within a few days. On examination, the visual acuity was 6/20 in the right and 20/20 in the left eyes, with a right relative afferent pupillary defect and decreased red color saturation. The fundus examination revealed optic disc swelling with venous dilation in the right eye and a normal left fundus with a crowded disc (disc-at-risk). The patient was evaluated for systemic disorders, all of which were normal. Findings were suggestive of non-arteritic anterior ischemic optic neuropathy most likely due to clomiphene.

**Conclusion:**

As clomiphene may increase blood viscosity, it is hypothesized that reduced flow in a posterior ciliary artery in conjunction with the disc-at-risk contributes to the anterior ischemic optic neuropathy. It is advised that patients with disc-at-risk be aware of the possible non-arteritic anterior ischemic optic neuropathy and those experiencing visual symptoms while taking clomiphene be examined promptly for evidence of optic nerve injury.

## 1. Introduction

Clomiphene citrate is an estrogen receptor ligand with mixed agonistic–antagonistic properties used for the treatment of female and male infertility (1). Clomiphene binds to the estrogen receptors on the hypothalamus, thus inhibiting normal estrogenic negative feedback and inducing testosterone production and spermatogenesis (2). Several visual side effects have been reported with increased dosage and duration of therapy with clomiphene (3, 4). A number of irreversible visual disturbances have also been associated with clomiphene citrate (5).

In this report, we present a patient with presumed clomiphene-induced optic neuropathy.

## 2. Case Presentation

A 33-yr-old man presented with decreased vision of the right eye referred to Amiralmomenin Hospital, Rasht, Iran in November 2018. His symptoms were not accompanied by any other ocular symptoms. The patient had no relevant family history or prior ophthalmological problems or head trauma/injury. He had no significant medical history, including diabetes mellitus and hypertension, and no history of smoking. He had been treated with clomiphene citrate 100 mg for 2 wk for infertility issues. The patient presented with a sudden decrease of visual acuity in the right eye on the 14${}^{\mathrm{th}}$ day of taking the treatment and subsequently developed complete loss of inferior visual field in a few days. He had no history of other medicine or herbal intake.

**Figure 1:**
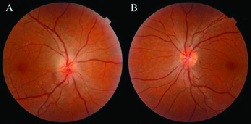
Fundus photograph: (A) Disc swelling and hyperemia in the right fundus and (B) A small cup-disc ratio in the left fundus.

At initial presentation, his best-corrected visual acuity was 6/20 in the right and 20/20 in the left eyes, with a right relative afferent pupillary defect and decreased red color saturation. The intraocular pressure was 13 and 11 mmHg in the right and left eyes, respectively. Extraocular movements were normal in both eyes. The slit-lamp examination of the anterior segment and anterior vitreous were unremarkable in both eyes. The fundus copy of the right eye revealed a swelling of the optic nerve head with blurred margin, especially in the superior pole pallor, and venous congestion was noted. The macula seemed to be normal. Fundus copy of the left eye showed a small cup–disc ratio of 0.2 without any other pathological findings (Figure 1). Based on these findings, immediate neurologic consultation was performed and brain and orbital MRI and computer tomography were obtained, which showed no pathologic findings. Cardiologic examinations including carotid ultrasound and blood pressure monitoring were also unremarkable. Further serologic studies for infection and hypercoagulable states (complete blood count, thyroid hormone levels, erythrocyte sedimentation rate, C-reactive protein, angiotensin-converting enzyme, toxoplasmosis antibodies, antithrombin III, factor V Leiden, protein C, protein S, anti-phospholipid antibody, and lupus anticoagulant) were evaluated. All laboratory tests were within the normal range.

**Figure 2:**
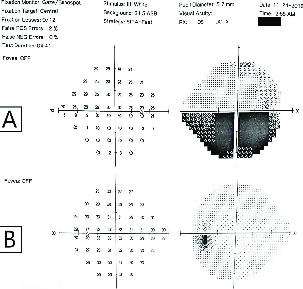
SITA Standard 24-2 Perimetry: (A) Right eye with an absolute inferior altitudinal visual field deficit and (B) A normal visual field in the left eye.

Perimetry by a Humphrey Visual Field Analyzer (24-2 threshold program) revealed an absolute inferior altitudinal visual field deficit in the right eye and a normal visual field in the left eye (Figure 2). Optical coherence tomography of the optic nerve showed thickening of the nerve fiber layer of the right eye and the macular optical coherence tomography revealed no significant changes.

Physical examination and complementary evaluations were suggestive of non-arteritic anterior ischemic optic neuropathy (NAION) of the right eye. Considering the normal laboratory and cardiologic findings and that the patient had been taking clomiphene citrate, it was concluded that clomiphene citrate was accountable for the anterior ischemic optic neuropathy. The patient was asked to discontinue clomiphene citrate. Three months later despite the drug discontinuation, the visual acuity was 10/20 in the right eye with a relative afferent pupillary defect and superior disc pallor, with a persistent inferior altitudinal visual field defect.

### Ethical considerations

The case report was fully explained to the patient and written consent was obtained to authorize the authors to write and publish a case report.

## 3. Discussion

NAION is a common optic neuropathy occurring generally in adults over 50 yr (6). Although not well-understood, several mechanisms have been mentioned regarding ischemia. Arteriosclerosis with or without thrombosis and embolization reduces blood flow of the short posterior ciliary arteries; however, it is believed that in some cases the condition is due to generalized hypoperfusion. The inflexible structure of the optic disc has also been associated with NAION, thus suggesting a compartment syndrome as a mechanism (7). Patients with a small cup–disc ratio, also known as disc-at-risk, are also prone to NAION. Nearly all cases have at least one vascular risk factor that contributes to NAION. A number of medications have been linked with the development of NAION including interferon-alpha, phosphodiesterase inhibitors, and amiodarone (6).

Clomiphene citrate is a nonsteroidal drug with both estrogenic and antiestrogenic activity (1). It is the treatment of choice for women with anovulatory infertility and has also been used for the treatment of male infertility. Reported side effects include headache, dizziness, nausea, vomiting, gynecomastia, hypertension, pancreatitis, myocardial infarction, deep vein thrombosis, pulmonary embolism, and hypertriglyceridemia (2). Increased dosage and duration of therapy with this drug has been associated with several visual side effects (8). Visual problems such as decreased vision, diplopia, photophobia, scotomata, periphlebitis, and retinal vein occlusions have been reported in the literature (4, 9–12). Clomiphene therapy causes the release of pituitary gonadotropins followed by an increase in thrombogenic estradiol which increases blood viscosity and the likelihood of blood to coagulate. The probable increase in blood viscosity and coagulation predispose patients to vascular-related incidents.

In the present case, it is hypothesized that reduced blood flow in a posterior ciliary artery in conjunction with the disc-at-risk contributed to the anterior ischemic optic neuropathy. Our patient was an otherwise healthy young man with no prior vascular risk factor and denied the use of any medication apart from clomiphene. To the best of our knowledge, two reports of clomiphene-associated NAION have been documented so far (13, 14). Similar to the present case, in both of these cases, clomiphene was the sole drug used and patients had no related underlying disease. Infertility specialties and ophthalmologists must be cautious of the possible optic nerve injury in connection with clomiphene citrate. Therefore, we suggest that before starting treatment, a careful ophthalmic examination should be carried out and patients with the disc-at-risk should be informed about the increased possibility of NAION occurrence. Also, patients experiencing visual symptoms while taking clomiphene must be examined promptly for evidence of visual changes or optic nerve injury.

## 4. Conclusion

As NAION is an irreversible cause of visual loss, special consideration must be given when prescribing clomiphene citrate. It is recommended that patients undergo ophthalmic examination prior to clomiphene initiation and those under treatment with clomiphene should be examined promptly if they experience visual disturbances.

##  Conflict of Interest

None declared.
